# Supplementary material for: Exercise Intervention Mitigates Pathological Liver Changes in NAFLD Zebrafish by Activating SIRT1/AMPK/NRF2 Signaling
Source: Int J Mol Sci. 2021 Oct 10;22(20):10940. doi: 10.3390/ijms222010940 (PMC8536011; doi:10.3390/ijms222010940)
Supplement: Supplementary file 1 [file ijms-22-10940-s001.zip › ijms-1403594-supplementary.pdf]

Table S1. The primers used in this study.

| Primer | Forward | Reverse |
|---|---|---|
| *β-actin* | GTCATCACCATCGGCAAT | CGTGGATACCGCAAGATT |
| *acaca* | CGGGCTCGAGACAAGTTTGA | GCTCCCAAGTACAGGTGCAT |
| *srebf1* | AGTTCTCCGACGCTCTTG | TGACCACCACCACCATTG |
| *fasn* | AAACTGCACGAGGTGTGTGA | ATGCGAAGGTTTAGCCCTCT |
| *pparg* | CTGCCGCATACACAAGAAGA | TCACGTCACTGGAGAACTCG |
| *pgc1α* | GCGAGGGAACGAGTGGATTT | CTCTCCACACCGAATCCTGA |
| *pparab* | ATGTCCCACAATGCCATCCG | TCTGCTTGGCCAGGGTTTTC |
| *cpt1* | TGGCACTGCAACTAGCTCAA | AACCTCTGCTCATTCGTGGT |
| *acox1* | TGGATCCCATTAGCTGAGTCATT | TTGAGGTTTTACCCAATCCACCT |
| *tnfα* | AGGAGAGTTGCCTTTACCGC | GTGAGTCTCAGCACACTTCCA |
| *il1β* | GATCCGCTTGCAATGAGCTAC | TCAGGGCGATGATGACGTTC |
| *capase3* | TCTACAATGACCAGACAGTTG | AAGACTTGAGATCCACAGATG |
| *bax* | GGCTATTTCAACCAGGGTTCC | TGCGAATCACCAATGCTGT |
| *bcl2* | CTTTCAAAGCGAGGATATGTG | CTATCAGGCATTCAGAGTTGT |
| *nqo1* | GCAGAATCCCGAGCACTTTG | TTCTTCTGCGATCAGCTGAAAG |
| *ho-1* | ACAGAGACTGAGAGAGATTGGC | TCTATTGGCGCTCGTCACTC |
| *cat* | CATCCAGAAACGCATGGTGC | ACGCTCCACCACGTGAATAA |
